# 

**Published:** 1894-10

**Authors:** 


A New Surgical Work.—A System of Surgery, edited by Frederic S. Dennis, M. D., and John S. Billings, M. D., is announced by Lea Brothers & Co. as shortly to appear in three imperial octavo volumes. The list of contributors embraces such names as W. T. Bull, Charles McBurney and Robert F. Weir, of New York; Councilman, Porter, Richardson and Warren, of Harvard; Carmalt, of Yale; Keen, White, Roberts and Wharton, of Philadelphia; Welch, of Baltimore; Park, of Buffalo; Conner, of Cincinnati; Mudd, of St. Louis; and Senn, of Chicago.

An Intra-Mural View, a very artistic brochure, has been received from the Curtis Publishing Company, Philadelphia, publishers of *The Ladies' Home Journal.* As the title indicates, the booklet gives us glimpses of the interiors of the *Journal's* offices and some idea of the work carried on there. The main building, entirely occupied by the editorial and business offices, was designed by Mr. Hardenbergh, the architect of the Hotel Waldorf, New York, and was completed in January, 1893. The exterior is attractive and the interior elegantly appointed and admirably planned. The numerous illustrations, showing the commodious and well-fitted offices, and the accompanying text, giving us some insight into the work in the different bureaus, requiring a force approximating 400 employees, indicate the wonderful success which *The Ladies' Home Journal* has achieved in an almost incredibly short time. The brochure also describes at some length the work of printing and binding the *Journal*, which is carried on in a separate building. An Intra-Mural View will be sent to any one who will address The Curtis Publishing Company, and inclose four cents in stamps for postage.

Notice to Contributors.—We are glad to receive contributions from every one who knows anything of interest to the profession. Articles designed for publication in the Journal should be handed in before the first day of the month. The Editors are not responsible for the views or opinions of contributors. All communications should be addressed to the Managing Editor, 284 Franklin St., Buffalo, N. Y.

